# Sustainable 4D Printable Biobased Shape Memory Polymers with Linear Tunability and Multistimuli Actuation for Advanced Applications

**DOI:** 10.1002/smsc.202500104

**Published:** 2025-04-29

**Authors:** Maksims Jurinovs, Madara Veseta, Alisa Sabalina, Pedro E. S. Silva, Artis Linarts, Hossein Baniasadi, Jaana Vapaavuori, Sergejs Gaidukovs

**Affiliations:** ^1^ Institute of Chemistry and Chemical Technology Faculty of Natural Sciences and Technology Riga Technical University P. Valdena Str. 3 Riga LV‐1048 Latvia; ^2^ Department of Chemistry and Materials Science School of Chemical Engineering Aalto University Kemistintie 1 02150 Espoo Finland; ^3^ Institute of Physics and Materials Science Faculty of Natural Sciences and Technology Riga Technical University Paula Valdena 3/7 Riga LV‐1048 Latvia; ^4^ Polymer Synthesis Technology School of Chemical Engineering Aalto University Kemistintie 1 02150 Espoo Finland

**Keywords:** 3D printing, actuators, additive manufacturing, plant‐based acrylates, sustainability

## Abstract

Sustainable materials that effectively combine sophisticated functionality with eco‐friendly materials are critical for next‐generation technologies. Herein, a novel, fully bioderived, 4D printable shape memory polymer with linear tunability and remotely controlled actuation capabilities is presented. Using a linearly tunable matrix based on plant‐derived acrylates with biosourced carbon content ranging from 75% to 87%, such as acrylated rapeseed oil, isobornyl acrylate, and isobornyl methacrylate, precise linear control over glass transition temperatures and mechanical properties is achieved. Furthermore, incorporating up to 0.2 wt% carbon nanotubes enhances electrical and thermal conductivity, enabling Joule heating and light‐driven activation of 4D‐printed actuators. These materials demonstrate remarkable shape fixity and recovery ratios above 90%, validated through thermomechanical analysis. Complex geometries, including auxetic and spiral structures, are successfully fabricated using vat photopolymerization 4D printing, highlighting exceptional resolution and defect‐free printing. Dual‐stage actuation and modular recovery capabilities are demonstrated for multifunctional applications. The materials reported here outperform conventional petroleum‐based acrylates, requiring significantly lower activation voltages while maintaining rapid and efficient recovery. Developed biobased systems open pathways for sustainable applications in soft robotics, aerospace, adaptive medical devices, and smart textiles, paving the way for greener technologies.

## Graphical abstract

Multistimuli‐responsive biobased shape memory polymers enable rapid, remotely controlled transformations. The plant‐based actuators with various activation pathways open the ability for programmable shape recovery, with stepwise actuations dictated by localized energy inputs. These sustainable smart materials offer reversible, selective actuation, paving the way for applications in soft robotics, biomedical devices, and adaptive textiles.

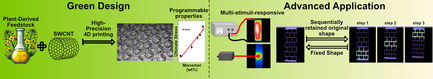

## 1 Introduction

The rapid evolution of smart materials and 3D printing technologies has stimulated advances in numerous fields, including robotics, biomedical devices, textiles, and adaptive surface engineering.^[^ ^1^, ^2^, ^3^ ^]^ Among these materials, shape memory polymers (SMPs) stand out for their unique ability to “remember” and return to a programmed shape when exposed to external stimuli.^[^ ^3^ ^]^ However, despite their vast potential, traditional SMPs have primarily relied on petroleum‐derived resources, limiting their renewability, biodegradability, and compatibility with sustainable practices.^[^ ^2^, ^4^, ^5^, ^6^ ^]^ As demand for eco‐friendly materials intensifies, there is an urgent need to develop SMPs that are fully bioderived yet capable of sophisticated shape memory behavior and activation pathways.

Biobased SMPs offer a compelling solution to conventional materials’ challenges, offering renewability, biodegradability, and lower environmental impact.^[^ ^6^, ^7^ ^]^ Recent research has made progress in developing biobased SMPs with tunable properties,^[^ ^8^, ^9^, ^10^ ^]^ but translating these materials into fully scalable, 3D‐printable actuators remains an ongoing challenge.^[^ ^11^ ^]^ The majority of the research available in the literature focuses on the 3D printing of thermoplastic polymers, such as poly(lactic acid), poly(butylene succinate), their blends, and several other biobased polyesters, using fused filament fabrication (FFF) technology.^[^ ^12^, ^13^, ^14^ ^]^ While more popular and easier to operate, FFF has a much lower resolution of developed structures, and developed systems are mostly only heat‐activated. Designing SMP that respond to multiple, low‐energy stimuli without compromising mechanical performance or printability presents both an opportunity and a considerable optimization curve.^[^ ^15^ ^]^ For example, Huang et al. developed a polylactic acid/epoxidized natural rubber/Fe_3_O_4_ biobased system, capable of multistimuli actuation by light, heat, and magnetic fields with shape recovery ratios above 95%.^[^ ^15^ ^]^ However, the developed polymer and samples were manufactured using conventional injection molding, strictly limiting the available structures and their complexity. Integrating these multistimuli features in a fully bioderived polymer system compatible with high‐precision vat photopolymerization 3D printing (VPP) would enable the development of sustainable, multifunctional actuators for a wide range of applications almost without geometry limitations.^[^ ^16^ ^]^ Such an approach will lead to the development of a novel class of sustainable materials, enabling 4D printing, where the fourth dimension—time—is integrated into 3D‐printed shape memory structures.^[^ ^17^ ^]^

Existing SMP technologies demonstrate impressive shape‐memory performance, but multistimuli responsiveness remains an underexplored frontier, especially in 4D printable biobased systems. While only temperature‐activated SMPs are common,^[^ ^10^, ^18^, ^19^ ^]^ polymers that can respond predictably to diverse stimuli at the same time while being also 4D printable remain underexplored. Several studies have been reported on diverse single‐stimulus petroleum‐based systems activated by Joule heating,^[^ ^16^ ^]^ solvents,^[^ ^20^ ^]^ magnetic fields,^[^ ^21^ ^]^ and light irradiation.^[^ ^22^ ^]^ Among these, Joule heating and light stimulus have several advantages, such as localized heating, rapid response, and remote control, allowing precise and targeted actuation.^[^ ^16^, ^22^ ^]^ Stimuli‐responsive materials hold immense potential for creating low‐energy, adaptive devices with real‐world applications, such as energy‐efficient actuators or autonomous medical devices.^[^ ^23^, ^24^ ^]^

Several strategies can be utilized to introduce multistimuli responsiveness in SMPs. For example, metallic fillers, such as Fe_3_O_4_, can help to introduce responsiveness to magnetic fields.^[^ ^25^, ^26^ ^]^ Carbonaceous fibers are also known to improve the performance of SMP, due to their improved thermal and even electrical conductivity.^[^ ^27^, ^28^, ^29^, ^30^ ^]^ Although carbon nanotubes (CNTs) have emerged as critical additives for expanding the application scope of SMPs,^[^ ^31^, ^32^, ^33^ ^]^ their high electrical conductivity, thermal stability, and mechanical reinforcement capabilities make CNTs ideal for introducing remote activation capabilities, such as Joule heating^[^ ^34^ ^]^ or light absorption,^[^ ^35^ ^]^ into SMPs. Despite these advantages, the integration of CNTs into polymers presents notable challenges. CNTs tend to aggregate during formulation due to their high surface energy, leading to heterogeneous dispersion and uneven performance across the material.^[^ ^36^, ^37^ ^]^ Furthermore, their incorporation into ultraviolet (UV)‐curable systems introduces additional challenges. UV‐curable resins, known for their rapid curing, low energy requirements, and compatibility with high‐resolution 3D printing, rely on photopolymerization processes that CNTs interfere with. CNTs absorb UV light, reducing the curing depth and potentially altering the materials’ crosslinked structure.^[^ ^36^, ^37^, ^38^ ^]^ These issues necessitate careful optimization of resin formulations and processing conditions. Obtaining tunable mechanical properties, precise control over shape recovery, and, in the case of UV‐curable materials, optimal curing for 4D printability within a single material has proven difficult, limiting available literature on the subject primarily to fossil‐based polymers. Only a few studies exist on CNT‐filled SMPs for VPP printing, underlining the multiple issues that need to be solved in this area.^[^ ^16^, ^38^, ^39^ ^]^

This work introduces the first case of a fully biobased VPP 4D printable SMP system designed to address these challenges, exhibiting controlled, multistimuli responsiveness to hot air, light, and electric fields. By incorporating plant‐based precursors with high biosourced carbon content (BSC), such as acrylated rapeseed oil (ARO) (87% BSC), isobornyl acrylate (IBOA, 75% BSC), and isobornyl methacrylate (IBOMA, 76% BSC) and optimizing the acrylate‐methacrylate ratios, we developed a linearly tunable bioderived matrix capable of remarkable shape fixity and recovery ratios of over 90%. The linear tunability of developed materials allows for precise control over mechanical properties and transition temperatures, making them compatible with advanced approaches to VPP 4D printing. Moreover, the addition of CNTs further expands the application scope by enhancing electrical and heat conductivity, creating a system adaptable to remote and direct activation. This study contributes to sustainable materials science and advanced manufacturing, presenting a novel, fully plant‐derived SMP capable of multistimuli via hot air, light, and electricity‐responsive 4D printing. By leveraging biobased precursors, we demonstrate the potential for environmentally responsible materials without compromising performance, opening new possibilities for soft robotics, adaptive medical devices, and sustainable actuator designs.

## 2 Results and Discussions

### 2.1 Optimization and Characterization of Fully Bioderived SMPs

Functionalized plant oils are known to enable shape memory behavior in cured polymers. Vegetable oils (VO) have been utilized in various epoxy or polyurethane systems, where differences in the crosslinking nature help to gain the ability to fix and hold particular programmed shapes.^[^ ^40^, ^41^ ^]^ IBOA or IBOMA have been utilized in combination with various petroleum‐based acrylates, hydroxyethyl methacrylate, and polyurethane acrylates, where the shape memory was mainly based on reversible hydrogen bonding.^[^ ^39^, ^42^, ^43^ ^]^ In this work, we aim to develop a fully bioderived shape memory system, combining it with VPP to boost the variability of the applications and shape complexities. All used acrylate polymers used in our work have high biosourced carbon content above 75%, and their structural formulas can be seen in Figure S1, Supporting Information. By incorporating ARO (A in sample names) fixed at 50 wt% and changing the IBOA (I in sample names) to isobornyl methacrylate (M in sample names) mass ratios from 1:1 to 1:7 (I:M), we were able to fine‐tune the mechanical and thermomechanical characteristics of the developed 3D printable polymers. The prepared polymers and their labels are shown in Table S1, Supporting Information. Furthermore, incorporating CNTs further enhanced the system's variability by enabling Joule heating and light‐responsive behavior, introducing the ability for a remotely controlled actuation.

**Figure 1:**
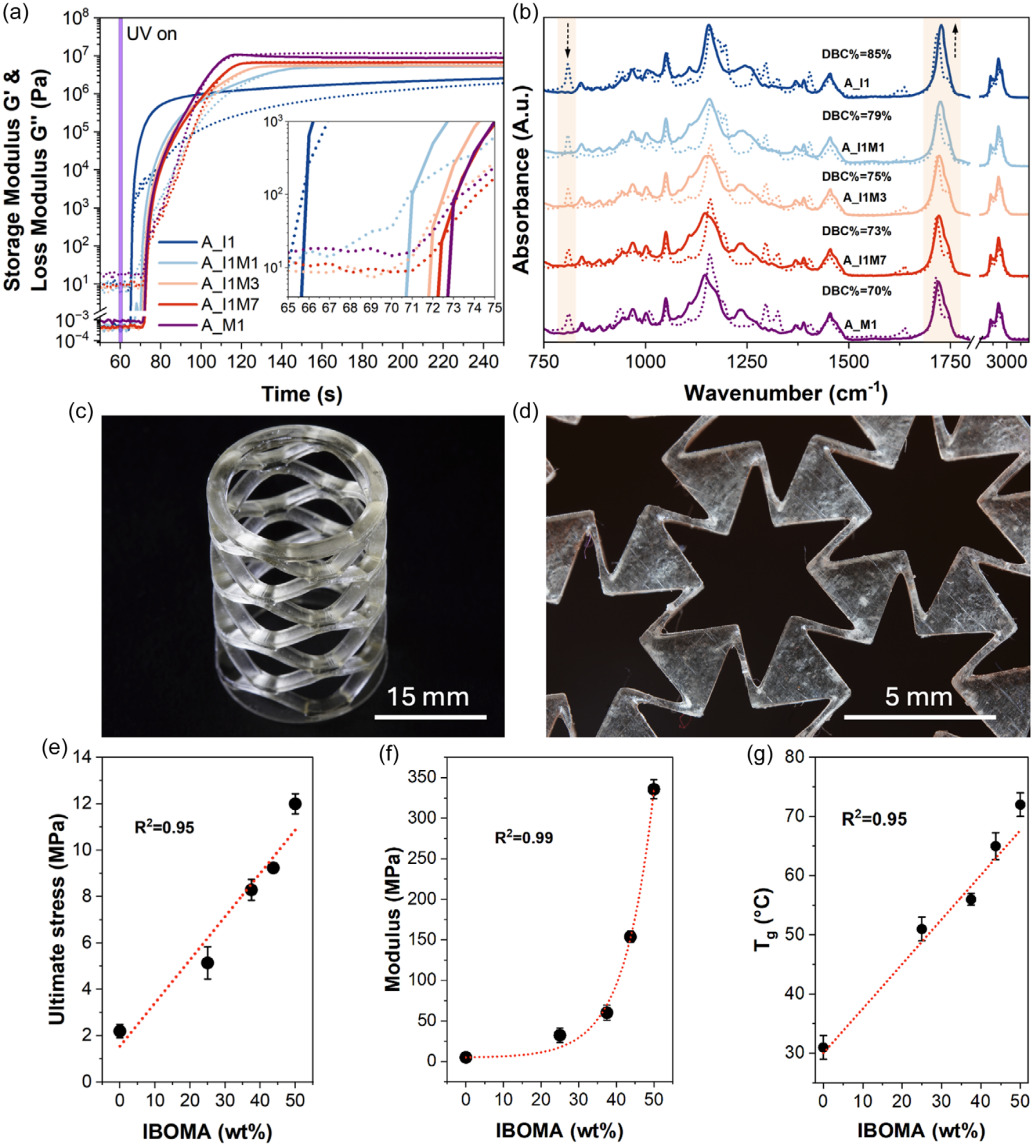
a) Photorheology plots showing the evolution of storage (solid lines) and loss modulus (dotted lines). b) FTIR spectra before (dotted lines) and after (solid lines) 4D printing, with calculated DBC% values for the bioderived polymer matrix. c) 4D‐printed intricate spiral structure and d) scaled‐down auxetic‐pattern structure, highlighting the exceptional accuracy of the developed resins. e) Fitting of ultimate (break) stress, showing a linear relationship. f) Fitting of tensile modulus, showing an exponential relationship. g) Fitting of the glass transition temperature (*T*_g_), showing a linear relationship for the 4D‐printed bioderived polymer matrix.

As the first step, the polymerization behavior and network formation of the biobased SMPs were examined with the use of photorheology and Fourier‐transform infrared spectroscopy (FTIR) (**Figure** **1**). The real‐time evolution of the storage modulus (*G*′) and loss modulus (*G*″) was recorded during UV‐induced curing for formulations with varying ratios of IBOA and IBOMA (Figure 1a). All formulations showed a rapid increase in *G*′ after UV irradiation, indicating crosslinked network formation. A_I1, containing the highest IBOA content, exhibited the highest curing rate, reaching a *G*′ of ≈10^6^ Pa within 20 s, suggesting highly efficient crosslinking due to the higher reactivity of IBOA. In contrast, A_M1, composed of ARO/IBOMA IN 50/50 ratio, showed slower curing kinetics, with the final modulus of ≈10^7^ Pa achieved in around 50 s. Photorheology results indicate that adding IBOMA leads to decreased curing kinetics, as all intermediate formulations (A_I1M1, A_I1M3, and A_I1M7) demonstrated progressively slower curing. As a result, the gelation time, which is identified by a crossover of the *G*′ and *G*″ and marks the onset of the formation of an interconnected polymer network, shifted from ≈5.5 s for A_I1 to ≈13 s for A_M1. The photorheology also gave insight into the mechanical parameters of the crosslinked network, revealing a strong positive correlation between storage modulus and IBOMA concentration. Such tendency is attributed to the inherently stiffer nature of the methacrylate backbone, which compensates for the lower conversion rate and reduced reactivity by forming a more rigid structure, increasing *G*′.^[^ ^44^, ^45^ ^]^ The double bond conversion values (DBC%, Equation (1)), calculated using the intensity of the vinyl bond stretching vibrations at ≈810 cm^−1^ and carbonyl group at ≈1730 cm^−1^, correlated well with the photo‐rheological data, where A_I1 showed the highest DBC% of 85%, confirming its high crosslink efficiency (Figure 1b). As the IBOMA content increased, the DBC% progressively decreased, reaching 70% for A_M1. The lower DBC% and higher gel times in IBOMA‐containing formulations reflect the slower reactivity and less efficient crosslinking of methacrylate monomers, as the steric hindrance of the methyl group reduces the accessibility of the double bonds for formed radicals during polymerization.^[^ ^44^, ^46^ ^]^

Considering the curing kinetics of the formulations, all samples (except composites, due to reasons discussed later) were printed using 20 s layer time. This amount of time guaranteed samples that were robust enough to be removed from the printing platform and handled quickly until further UV‐postcuring for 5 min in the UV chamber. It was essential to remove the samples from the printing platform prior to postcuring; if they were not, they adhered to the platform too firmly and were impossible to remove without breaking. As a result, we successfully 3D‐printed several structures to demonstrate the capability of the developed materials in producing highly detailed and complex geometries. Figure 1c shows a spiral structure with numerous overhangs, which typically challenge material performance due to the stresses caused by adhesion to both the printing platform and the vat surface. Figure 1d presents a scaled‐down auxetic structure with joints as small as 250 μm, where fragility often leads to breakage during postprocessing. Remarkably, both structures were printed without defects, highlighting the exceptional mechanical robustness, printability, and resolution achieved by the optimized materials in demanding geometries.

Based on the photorheology insights, further tests focused on the mechanical and thermomechanical performance of 3D‐printed samples. The tensile tests (Figure S2a, Supporting Information) confirmed a clear correlation between the IBOA/IBOMA ratio and mechanical performance, which was indicated by photorheology results. The mechanical strength and modulus (Figure 1e,f) of the 4D‐printed polymers increased gradually with the increase of IBOMA content, with highest values of 13 and 325 MPa for A_M1 and lowest of 2.3 and 10 MPa for A_I1 sample, respectively. Furthermore, the ultimate (break) stress fitting in Figure 1e showed a strong linear correlation with the concentration of IBOMA. This correlation allows for easy and predictable tuning of the mechanical performance of the developed materials by simply varying the IBOA/IBOMA ratios.

Conversely, Figure 1f illustrates the critical role of the IBOMA in obtaining the crosslinked network's rigidity, as the tensile modulus increases exponentially with the addition of the latter. At low IBOMA concentrations, the modulus increases slowly as the methacrylate‐rich domains are isolated within the softer, elastic ARO and IBOA matrix. Several reports show that careful macromolecular network design can help develop UV‐curable elastomers using IBOA, urethane diacrylates, or acrylated VOs.^[^ ^47^, ^48^ ^]^ However, as the IBOMA content increases, the methacrylate segments form continuous, interconnected regions within the polymer network, increasing rigidity and resulting in a sharp increase in modulus. This theory is backed by the characteristic deformation curve changes, indicating a transition from elastic to plastic deformation, attributed to a much more rigid crosslinked network.

Dynamic mechanical analysis (DMA), performed on 4D‐printed films, revealed that the storage modulus (*E*′) confirms the tendency observed previously; higher IBOMA content leads to increased stiffness, as indicated by the higher storage moduli across the temperature range, with A_M1 exhibiting the highest *E*′ at room temperature (Figure S2b, Supporting Information). The tan *δ* peaks (Figure S2b, Supporting Information), representing the glass transition temperature (*T*_g_), progressively shift to higher temperatures with increasing IBOMA content, confirming a linear relationship between IBOMA content and *T*_g_ (Figure 1g). The tunability of *T*_g_ through compositional control is significant for applications requiring shape memory effects, as the transition temperature largely determines the thermal response of the material.^[^ ^49^ ^]^ The ability to adjust *T*_g_ from ≈32 °C (A_I1) to 72 °C (A_M1) allows for precise control over the activation temperatures for shape memory behavior. The sharp decrease in storage modulus post‐*T*_g_ (observed for all samples) suggests a relatively rapid transition from a rigid to a soft, rubbery state, critical for efficient shape recovery in shape memory applications. For SMPs, this feature ensures that once heated above *T*_g_, the material can quickly switch back to its original configuration from a temporarily fixed shape.^[^ ^49^, ^50^ ^]^

### 2.2 Characterization of Fully Bioderived Shape Memory CNT Nanocomposites

Because of light absorption and blocking, especially at higher loadings, it is well‐known that incorporating CNTs can lead to decreased mechanical performance and slower curing rates.^[^ ^16^, ^36^, ^37^ ^]^ Based on the material optimization insights, we prepared the CNT composites using the A_I1M7 formulation. This formulation exhibited a DBC% higher than systems using only ARO/IBOMA and demonstrated superior tensile modulus and stress compared to formulations with lower IBOA/IBOMA ratios while maintaining high elongation. The rationale behind this choice was to mitigate the potential detrimental effects of CNTs on the curing process. However, as the subsequent analysis will reveal, additional postprocessing steps were still necessary to achieve optimal performance. Table S1, Supporting Information, shows the final compositions of the composite formulations. The numbers indicate the mass percent of CNTs, which were 0.1 and 0.2 wt%, while letter *T* indicates whether the sample was thermally postcured to achieve optimal cross‐linking.

Incorporating particles into VPP resins inevitably increases viscosity, which becomes a critical limiting factor for successfully recoating the vat surface, directly affecting the material's ability to be 3D printed.^[^ ^38^ ^]^ As expected, the viscosity curves in Figure S3, Supporting Information, indicate a substantial increase in viscosity for composite resins. The CNTs led to a typical shear‐thinning behavior of the resins, although numerous reports in the literature show that viscosity values of <10 Pa.s at low shear rates (<10 s^−1^) are generally suitable for successful VPP printing. At the same time, higher viscosities demand several modifications of the process.^[^ ^44^, ^51^ ^]^

**Figure 2:**
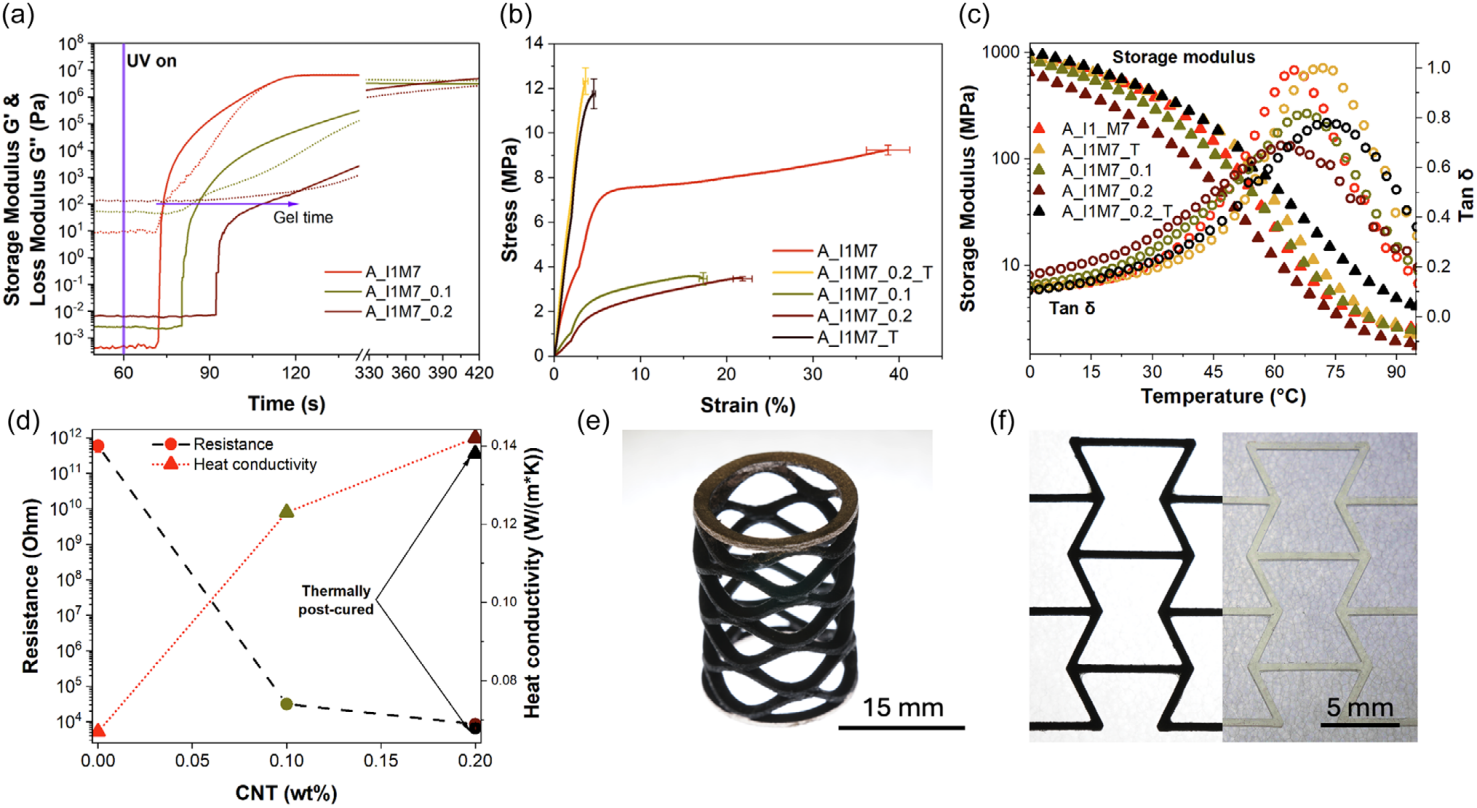
a) Photorheology plots illustrating the evolution of storage (solid lines) and loss modulus (dotted lines) during neat and composite resin curing. b) Stress‐strain curves and c) storage modulus and Tan*δ* curves of 4D‐printed samples. d) Resistance and heat conductivity changes with an increase of CNT concentration at 20 °C. e) A 4D‐printed composite spiral structure and f) comparison of auxetic composite (left) and neat (right) polymer structures, highlighting the exceptional accuracy of the composite resins.

As the next step, we addressed the UV‐curing challenges of particle‐filled systems. The addition of CNTs into the A_I1M7 bio‐derived matrix resulted in distinct changes in the material's rheological and mechanical properties. As evidenced by the delayed rise of *G*′ in **Figure** **2**a, CNTs hinder the curing process of the composite resins. The higher the CNT concentration, the higher the gel time, with the neat matrix's gel time of ≈12 s, and increase to ≈36 s for 0.2 wt% CNT composites. In addition, the development of *G*′ shows that all formulations can achieve similar values of 10^6^–10^7^ Pa, demonstrating that CNT‐filled composites can achieve mechanical performance similar to the neat polymer over a much longer curing time. However, the light‐penetration depth becomes a limiting factor in this case, which can cause under‐curing, even with extended UV postcuring time.^[^ ^36^, ^52^ ^]^ This was observed later in the tensile and DMA tests. As a result, composites printing time was adjusted to 60 s for 0.1 wt% and 90 s for 0.2 wt% CNT, with UV postcuring extended to 10 min to ensure higher crosslinking. The DBC% values in Figure S4, Supporting Information, reveal that even with extended curing times, the crosslinking density of the CNT composites remained significantly lower, 68% for 0.1 wt% CNT and 56% for 0.2 wt% CNT. To evaluate the ability to print intricate structures using composite resins, the formulation with the highest CNT concentration was selected, as preliminary studies showed it exhibited the lowest mechanical properties among all tested compositions. The spiral structure shown in Figure 2e demonstrates that increased curing time provided sufficient mechanical integrity to withstand the pulling forces during the printing process. Moreover, Figure 2f showcases the exceptional printing accuracy of the composite resin, with no observable defects in the printed structures.

Figure 2b clearly illustrates the observations previously described; the CNT composites exhibit much lower mechanical integrity. Due to the increased thickness of tensile samples, light cannot fully penetrate the entire sample volume, resulting in highly undercured samples with low modulus and stress values even after increased UV postcuring times. A rheological approach employing high‐precision normal force measurements was adopted to demonstrate the decrease in curing depth of CNT composite resin.^[^ ^36^, ^53^ ^]^ Figure S5a, Supporting Information, illustrates that the neat A_I1M7 resin can be fully cured even with a 1 mm curing layer. In contrast, the A_I1M7_0.2 composite resin, when UV irradiated for the same amount of time (140 s), fails to achieve the gel point. Figure S5b, Supporting Information, shows that the total cured layer thickness is 310 ± 13 μm. The trend is evident: Higher CNT concentrations decrease all mechanical parameters of the samples, leading to a twofold decrease in all characteristics. The same logical shift is observed in the tensile modulus in Figure S6, Supporting Information, where with increased CNT content, the modulus decreases from 153 MPa for neat A_I1M7 to only 36 MPa. Due to this, the sample with the highest electrical conductivity (Figure 2d) was chosen for further thermal postcuring, as due to heat ignorance of the material dimensions and opacity, such post‐treatment will substantially improve the mechanical integrity of the composite.^[^ ^54^, ^55^ ^]^ It is seen that samples with 0.2 CNT expressed the lowest resistance of around 8 ± 1 kΩ, while postcured samples further improved it to 6.5 ± 0.8 kΩ. The decrease is related to stress relaxation and further crosslinking of the material, resulting in increased synergy between the polymer matrix and CNTs.^[^ ^56^ ^]^ Figure S7, Supporting Information, shows that all samples indicated a negative temperature coefficient. Consequently, thermally treated samples performed almost identically to the neat postcured polymer matrix in terms of mechanical performance, resulting in a 3.3‐fold and 10‐fold increase in stress and modulus of A_I1M7_0.2_T compared to only UV‐cured sample (Figure 2b and S6, Supporting Information). This indicates that the incorporated CNTs were homogeneously dispersed in the resin, and the primary limiting factor of lower mechanical performance was the low DBC% (Figure S4, Supporting Information). The Cole–Cole diagram in the supplementary Figure S8, Supporting Information, backs up this claim, as the only outlying curve belongs to the A_I1M7_0.2 sample. The curve shape in the Cole–Cole plot allows us to detect inhomogeneities of the composite systems when its shape changes from a semicircle, indicating imperfections in the samples.^[^ ^57^ ^]^

The DMA in Figure 2c revealed that the modulus of A_I1M7_0.2_T is the highest throughout the temperature range due to the influence of CNT, which hinders the molecular mobility and thus leads to higher mechanical integrity of the samples.^[^ ^58^ ^]^ This also explains the slightly higher *T*_g_ (72 vs. 76 °C) of the thermally postcured 0.2 wt% CNT‐filled sample compared to neat thermally postcured samples. In addition, the introduction of CNT particles showed a twofold increase in thermal conductivity, meaning the ability for faster heating and potentially faster shape‐recovery times (Figure 2d). Interestingly, the thermally postcured sample with 0.2 wt% CNT exhibited slightly lower thermal conductivity at room temperature than the UV‐cured sample, although it rose as the temperature increased (Figure S9, Supporting Information). The dense, rigid network initially restricts phonon movement, the primary heat transfer mechanism in polymers, resulting in reduced conductivity at lower temperatures. As the temperature approaches *T*_g_, the crosslinked network begins to relax, facilitating better phonon transfer and thus increasing thermal conductivity.^[^ ^59^ ^]^

### 2.3 Hot Air, Electricity, and Light Actuated Shape Memory Effect

**Figure 3:**
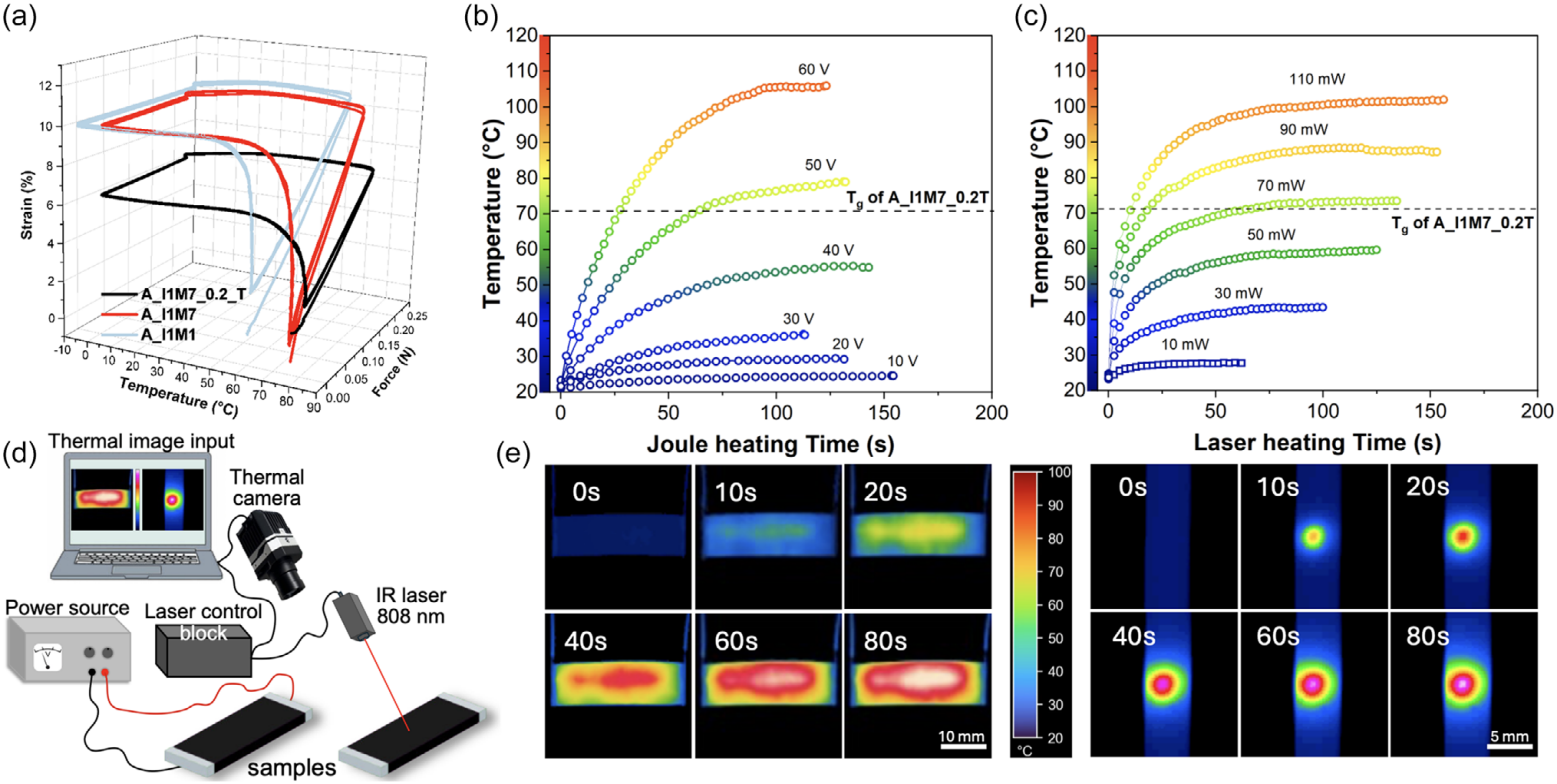
a) Representative shape memory cycles for samples further used for SMP structures 4D printing, demonstrating shape recovery and fixation of the 4D‐printed samples. The samples are first heated to *T* > *T*_g_, stretched, and then cooled to *T* < *T*_g_. Based on the residual strain, shape fixity is evaluated after the force is removed. Subsequently, the samples are reheated, and shape recovery is assessed. b) Maximum temperature variations during Joule heating of A_I1M7_0.2_T at increasing applied voltages. c) Maximum temperature variations during light irradiation of A_I1M7_0.2_T under increasing laser power (808 nm). d) Experimental setup for thermal imaging of SMP samples. e) Thermal images of samples heated by electricity (left, 60 V) and light (right, 110 mW).

Before moving on to 4D‐printed structures, first, we characterized the shape memory effect of all prepared bioderived materials. For this, thin 3D‐printed strips were analyzed using DMA in a typical shape memory effect cycle shown in **Figure** **3**a. All samples were evaluated for shape recovery (*R*_r_, Equation (2)) and shape fixity (*R*_f_, Equation (3)), with results summarized in Table S2, Supporting Information. The cycle graphs for each formulation are shown in Figure S10, Supporting Information. All formulations achieved *R*_f_ and *R*_r_ values exceeding 98% and 96%, respectively, demonstrating excellent shape memory performance comparable to fossil‐based VPP‐printed materials reported in the literature.^[^ ^4^, ^16^, ^60^ ^]^ Further tests assessed the applied voltage and laser power effect on the composite samples’ temperatures and heating rates. The air heating effect was not studied closely, as it is a straightforward process with minimal implications. The schematic experiment setup is shown in Figure 3d.

Expectantly, the increased CNT concentration and decreased resistance lowered the voltage and time needed to achieve temperatures above *T*_g_ of the CNT composites (Figure S7b, Supporting Information). Based on this and enhanced mechanical performance, the A_I1M7_0.2_T sample was used for electrically and light‐activated SMP 4D printing. Figure 3b demonstrates that applying a voltage of 50 V is sufficient to heat the sample above *T*_g_, while increasing it to 60 V accelerates this process to ≈40 s, enabling faster heating and, thus, actuation. Thermal imaging (Figure 3e and Video S1, Supporting Information) highlights the temperature distribution during Joule heating, where the center of the sample heats more rapidly than the edges due to ambient air effects and heat dissipation. Similarly, preliminary tests using an 808 nm laser with powers ranging from 10 to 110 mW revealed effective localized heating (Figure 3c and Video S2, Supporting Information). A laser power of 70 mW was sufficient to reach *T*_g_, but increasing the power to 90 and 110 mW reduced the heating time from ≈60 to ≈25 and ≈15 s, respectively. As shown in Figure 3e, the highest temperature is concentrated at the laser's focal point (≈1 mm diameter), with a gradual decrease in surrounding areas; thus, for rapid and efficient heating, laser power of 110 mW was utilized. In addition, a linear relationship between sample temperature and applied laser energy was observed, allowing for precise control of this characteristic (Figure S11, Supporting Information). Video S3, Supporting Information, demonstrates that the ability to heat material is achieved solely by incorporating black CNT particles into a bioderived matrix, as neat polymer does not heat up even at 110 mW.

**Figure 4:**
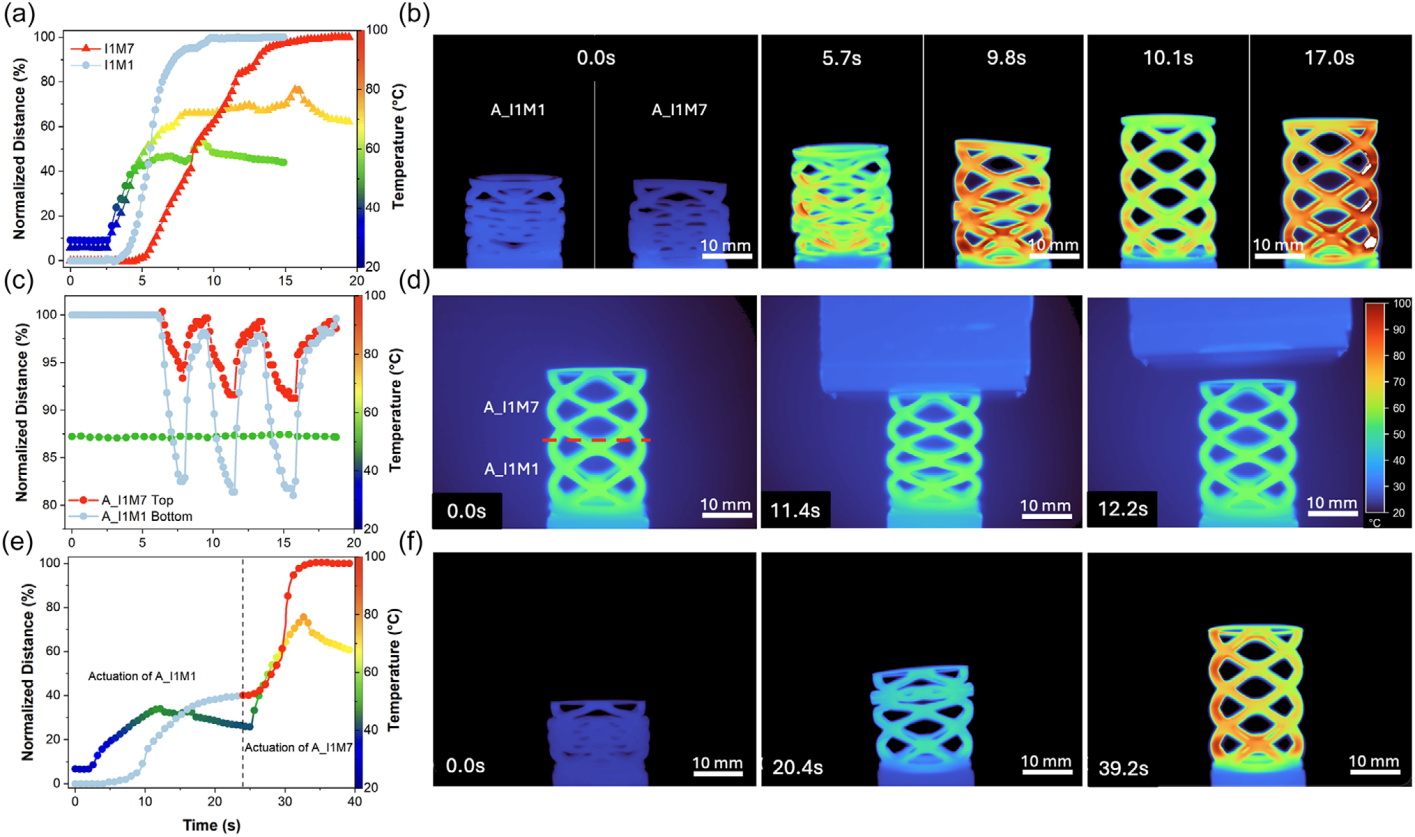
a) Shape memory effect and temperature of A_I1M1 (circles) and A_I1M7 (triangles) as a function of time and b) thermal imaging time‐lapse showing the shape memory effect of A_I1M1 (left) and A_I1M7 (right). Samples were heated with a hot air gun to *T* > *T*_g_ to activate the actuators. Due to higher rigidity and limited molecular mobility, A_I1M7 exhibited a slower shape memory response. c) Deformation behavior of a dual‐material structure fabricated via vat exchange (A_I1M1 at the bottom, A_I1M7 at the top) and d) thermal imaging time‐lapse of the dual‐material structure's response to applied force at 50 °C, applied by placing a 130‐gram hollow metal cube on top of the sample. e) Shape memory effect graph of the dual‐material 4D‐printed structure and f) thermal imaging time‐lapse, demonstrating sequential two‐step actuation: first, heating the sample to *T*_g_ (≈52 °C) of A_I1M1 to activate the softer material, followed by activation of the more rigid material by heating to *T* > *T*_g_ (≈65 °C) of A_I1M7.

We 4D printed several complex‐structure models to demonstrate the shape memory effect of the developed biobased polymers and composites. **Figure** **4**a,b (Video S4, Supporting Information) illustrates the distinct behavior of 4D‐printed spiral structures fabricated with two thermomechanically distinct materials: A_I1M1 and A_I1M7. These materials exhibit different actuation speeds at identical heating rates (to a higher temperature for A_I1M7 due to its elevated *T*_g_). The softer A_I1M1 spiral, with a *T*_g_ of ≈52 °C, activates and regains its original shape in ≈10 s. This rapid response is attributed to the lower activation temperature, which facilitates faster relaxation of the crosslinked molecular network. In contrast, the A_I1M7 structure, characterized by a higher methacrylate concentration, requires higher temperatures and longer times to achieve actuation. This delay results from the steric hindrance introduced by methacrylate groups, which restrict molecular mobility. Consequently, the A_I1M7 spiral takes around 17 s to regain its shape.

To further explore the versatility of our system, we 4D printed a hybrid spiral structure combining both materials within the same model. The bottom half was printed with A_I1M1, while the top half was printed with A_I1M7, using a simple vat exchange process previously reported by several authors.^[^ ^61^ ^]^ Figures 4c,d (Video S5, Supporting Information) illustrates the mechanical response of the dual‐material structure when subjected to an external force at ≈52 °C (*T*_g_ of A_I1M1). The softer A_I1M1 region deforms ≈2.5 times more than the harder A_I1M7 region, demonstrating differential mechanical responses based on material composition. This unique property enabled sequential activation. As shown in Figure 4e and thermograms in Figure 4f (Video S6, Supporting Information), the hybrid spiral undergoes a dual‐step deformation process. First, heating the structure to the *T*_g_ of A_I1M1 induced shape recovery in the bottom section of the spiral. Following a short cooling period, effectively demonstrating intermediate shape fixation, heating the structure to a temperature exceeding the *T*_g_ of A_I1M7 triggered the second shape memory stage. This step resulted in the complete activation of the spiral, showcasing the dual‐step actuation of the hybrid 4D‐printed material.

These findings highlight the potential of combining distinct biobased shape memory materials in a single 4D‐printed design to achieve tailored, multistage actuation. Such dual‐responsive behavior opens paths for advanced applications, including soft robotics, deployable structures, and programmable medical devices.^[^ ^60^, ^62^, ^63^ ^]^

**Figure 5:**
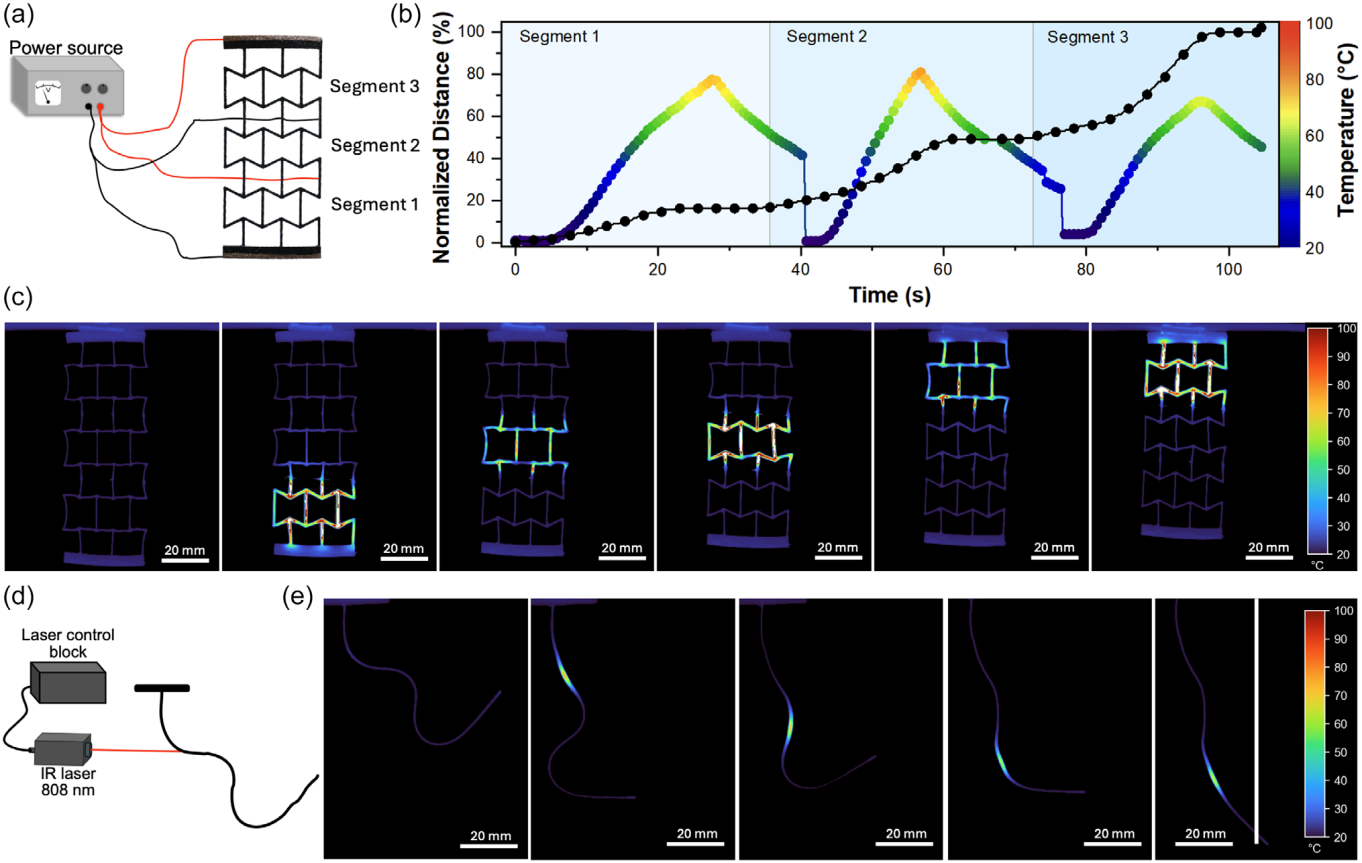
a) Experimental setup for sequential activation of a 4D‐printed auxetic structure. Thin copper wires were attached at specific positions on the model, with a silver coating applied to improve electrical contact. b) Shape memory effect and temperature evolution of the A_I1M7_0.2_T sample as a function of time (temperature drops after each segment appears due to setup reconfigurations). c) Thermal imaging timeline of the 4D‐printed auxetic structure sequential activation under 60 V. d) Experimental setup for light‐responsive shape‐memory actuators and e) thermal imaging timeline of the light‐actuated 4D‐printed A_I1M7_0.2_T, activated in seven consecutive steps to enable sequential segment activation.

As the next step, to showcase the ability of multistimuli activation, we fabricated and 4D‐printed an auxetic structure capable of easy, remotely controllable sequential activation of shape recovery with electricity. For this, copper wires were attached to four specific points on the structure, effectively dividing it into three distinct segments for stepwise actuation (**Figure** **5**a). The modular recovery process was observed after applying 60 V to each segment sequentially, which led to the Joule heating of the structure segment, initiating the shape recovery process (Figure 5b and thermograms in Figure 5c; Video S7, Supporting Information). Figure 5b shows the temperature increase immediately after voltage is applied to each segment of the structure, along with the corresponding time required for each segment to fully recover its original shape. Each segment achieved full recovery within ≈30 s, with the recovery time primarily limited by Joule the heating rate of the sample. The consistent recovery times across all segments highlight the homogenous distribution of CNTs throughout the structure. Notably, the applied voltage required for activation was significantly lower compared to petroleum‐based counterparts reported in the literature. For instance, fossil‐based VPP‐printed polymers with 2.5 times higher CNT content required ≈170 V for activation—a nearly threefold increase compared to the 60 V needed for developed biobased polymers.^[^ ^16^ ^]^ While the improved Joule heating efficiency is related to a complex interplay of polarity, viscosity, and crosslinking of the polymer matrix, this remarkable efficiency underscores the potential of these sustainable materials for applications demanding reduced energy input and enhanced actuation performance. The one‐step actuation of the printed structures is also achievable and is limited in speed only by the heating rate, as demonstrated in Video S8, Supporting Information. At the same time, interestingly, the A_I1M7_0.2_T sample exhibits faster hot air‐driven actuation compared to A_I1M7, achieving complete recovery in ≈10 s (Video S9, Supporting Information). This behavior can be attributed to the higher thermal conductivity of the CNT composites, which enhances heat transfer within the sample, thereby accelerating the shape recovery process.

However, it is important to note that while direct comparisons between samples with identical geometries are valid, comparing recovery times across different structures is not feasible. This is due to the interplay of multiple factors, such as geometry, heat dissipation, and structural complexity, significantly influencing the recovery process.

Finally, as a proof of concept, we conducted an experiment demonstrating light activation of a 1 mm thick, 90 mm long A_I1M7_0.2_T 4D‐printed bar. In this experiment, the laser was directed at specific locations on the sample to locally heat it to the activation temperature and induce shape recovery, as illustrated in Figure 5d. The precision of laser positioning was critical to the success of actuation, given the small laser spot size, which limited the heated area and, consequently, the efficiency of the overall recovery process. Despite this limitation, the thermograms in Figure 5e and Video S10, Supporting Information, demonstrate the successful recovery of the original shape of the sample. The entire recovery process took ≈10 min. This duration, however, can be significantly reduced by optimizing the laser spot size and placement to enable more uniform and effective heating tailored to specific application scenarios.

## 3 Conclusion

In this study, we demonstrated novel fully bioderived polymer matrices developed by combining plant‐based derivatives such as ARO, IBOA, and IBOMA, as a versatile, sustainable material for 4D printing of SMPs. Following the integration of CNTs, we developed composites with exceptional shape memory behavior and high‐precision 3D printability, and we gained the ability to achieve rapid multistimuli responsiveness. The successful 4D printing of complex geometries further underscored their potential for applications requiring dynamic shape changes and programmable functionality.

By optimizing the material compositions, we achieved linear activation temperature adaptation, ranging from 32 to 76 °C, with ensured exceptional shape fixity and shape recovery ratios up to 99%. Additionally, we could tune the mechanical performance of the developed materials in a range from 2.3 to 12.5 MPa in the same linear dependence. Moreover, overcoming challenges related to CNT incorporation, such as reduced curing and mechanical characteristics, enabled electrical and light‐based actuation of the 4D‐printed SMP composites. This provided a way for localized and precise control of the shape memory effect, offering distinct advantages over traditional activation techniques, such as plain immerging of the samples in hot water and hot air actuation.

Our findings make a significant step toward environmentally friendly, biobased alternatives to petroleum‐based materials, paving the way for sustainable materials in advanced applications. Further optimization and exploration of these materials could open new advancements in responsive systems for soft‐robotics, medical, and industrial applications, where shape‐changing capabilities and environmental sustainability are critical. Additionally, future research should address degradation and recycling strategies to tackle the inherent challenges of thermosetting, crosslinked systems.

## 4 Experimental Section

4.1

4.1.1

##### Materials

Unrefined rapeseed oil (RO) was purchased in a local supermarket. n‐Hexane, boron trifluoride diethyl etherate (BF3·OEt2), NaHCO3, acrylic acid, and Na_2_SO_4_, 2,2‐Azobis(2‐methyl‐propionitrile) (AIBN) were supplied by Sigma–Aldrich. The photoinitiator (PI) ethyl(2,4,6‐trimethylbenzoyl)phenylphosphinate (TPO‐L) was supplied by Fluorochem. IBOA (SARBIO 5102) and IBOMA (SARBIO 6105) were kindly supplied by Arkema. Single‐walled CNT (SWCNT), 1.6 ± 0.4 nm in diameter and ≥5 μm long were purchased from Tubal and used as received.

##### ARO Synthesis

Rapeseed oil synthesis route was adopted from a methodology reported in the literature.^[^ ^64^ ^]^ Briefly, rapeseed oil (RO) and acrylic acid were combined in a 1 to 0.5 (w/w) ratio. BF3·O·Et2 was added to the initial mixture in a 1 to 15 (w/v) ratio and stirred at 80 °C for 5 h. The cooled‐down mixture was dissolved in n‐hexane and rinsed with saturated NaHCO_3_ and NaCl solutions. The organic layer was separated from the water layer and was dried over anhydrous Na_2_SO_4_. After filtration, the solvent was removed using a rotary evaporator. The resulting ARO was used to prepare UV‐curable biobased resins by mixing them with IBOA, IBOMA, AIBN, and CNT in the ratios shown in Table S1, Supporting Information. The structural formulas of the bioderived acrylates with their bour sourced carbon content are shown in Figure S1, Supporting Information.

##### Sample Preparation and 3D Printing

Firstly, all resin parts were magnetically mixed for 1 h at desired ratios, and then 2 wt% of TPO was used as a photoinitiator. For the conductive composites, the desired amount of SWCNT was added to the mixture and ultrasonicated for 15 min in an ice‐water bath to prevent overheating and curing of the resin. During this process, high amounts of organic solvents are frequently used to improve the homogenization of the CNT within the composite. In this work, we avoided solvents during this step to benefit eco‐friendliness. Finally, the resulting resin was homogenized with a high‐shear mixer (Silverson L5M‐A, 5000 rpm) for another 20 min to improve the SWCNT dispersion in resins. For thermally postcured samples, 1 wt% of AIBN was added to formulations, during the mixing process. All samples were printed the next day after the preparation of the resin. A liquid sample of every resin was kept in a sealed container for over a month. During storage time, no separation or sedimentation was observed. The basis of resins that made up 100% of the composition were ARO, IBOA, and IBOMA. All additional components were added to the original composition.

Elegoo Mars 2PRO (M2P) digital light processing (DLP) printer was used to 3D print the samples. The printer utilizes a UV‐light‐emitting diode (LED) wavelength of 405 nm and a 6‐inch monochrome LCD screen with a 2560 × 1440 pixel resolution. Printing parameters were set with a layer height of 50 μm and a layer time of 20 s for neat samples and 60 (0.1 wt%) to 90 (0.2 wt%) seconds for the CNT composites

Following printing, the samples were rinsed in propanol and dried for 15 min at 37 °C. Afterward, all samples were postcured using a Prusa postcuring and washing machine (CW1, PRUSA Research, Czech Republic) for 5 min for neat resins and 10 min for composites. This machine employs four UV‐LED strips with a wavelength of 405 nm and a maximum power of 52.8 W. Thermally postcured samples were cured for 60 min at 140 °C. The dimensions of the printed samples differed depending on the specific testing requirements and are detailed in the descriptions of respective experimental methods.

##### Rheology

For photorheology measurements, an MCR302 rheometer from Anton Paar (Graz, Austria) was utilized. The rheometer had a plate/plate measuring system, a Peltier‐controlled temperature chamber with a 38 mm glass plate, and an 8 mm diameter metal top plate. Using a UV‐visible (UV‐Vis) spot curing system, OmniCure S1500 (Excelitas Technologies Corp., USA), the resin samples were exposed to radiation with the measurement gap set to 0.2 mm. Radiation was applied at room temperature (22 °C) at wavelengths ranging from 250 to 450 nm, with an intensity of 2 mW cm^−2^. The testing was performed in dynamic oscillatory shear time mode with an angular frequency of 5 rad s^−1^% and 1% oscillatory strain. The UV‐Vis onset was set at 60 s. Real‐time photorheology measurements continuously recorded storage modulus (*G′*) and loss modulus (*G″*).

A plate–plate‐shaped spindle with a 25 mm diameter was used to measure the viscosity of the resin. The testing was conducted in the shear rate range of 0.01–100 s^−1^ at 22 °C, with a 0.2 mm gap.

The curing depth of the selected neat resin and the CNT composite resin with the highest filler concentration was determined using a rheological approach described in the literature.^[^ ^53^ ^]^ First, a thick resin layer (500–1000 μm) was photopolymerized for 140 s, and the changes in modulus values were recorded. A storage modulus of 10^5^ Pa was used as the criterion for a fully cured, self‐supporting layer, confirming that the A_I1M7 sample was fully cured across the 500 and 1000 μm gaps. Since the cured layer thickness equaled the rheometer plate gap, further measurement was deemed unnecessary. In contrast, the A_I1M7_0.2 sample exhibited significantly lower modulus values, indicating incomplete curing. Subsequently, the upper plate was raised to remove the uncured resin. Next, the plate was lowered at a speed of 5 μm s^−1^, and the cured layer height was determined by monitoring the normal force versus gap. The cured layer thickness was defined as the gap at which a normal force of 2 N was recorded.

##### FTIR Spectroscopy

The interactions of the printed and raw resins were determined with an FTIR (Nicolet 6700, ThermoScientific, Germany). A fragment of the 3D‐printed biobased material, or a droplet of the raw resin, was placed directly on a diamond crystal and held with constant pressure using a torque wrench already attached to the spectrometer. The spectrum was obtained as an average of 16 separate spectra recorded in the 400–4000 cm^−1^ range with a resolution of 4 cm^−1^. Equation (1) was used to calculate the double bond conversion rate (DBC%).

(1) $$\text{DBC\%}=\left(1-\frac{\frac{A_{t}}{A_{\text{ra}}}}{\frac{A_{0}}{A_{\text{rb}}}}\right)\times 100$$

*A*_0_ and *A*_t_ are the C=C bond absorption intensity (810 cm^−1^) before and after polymerization, and *A*_rb_ and *A*_ra_ are the ester C=O bond absorption intensity (1722 cm^−1^) before and after polymerization.

##### Tensile Testing

A 25ST universal testing machine (Tinius Olsen, UK) ran tensile tests on 3D‐printed bio‐based dog‐bone (ISO 527‐1BA) shaped samples with dimensions 75 mm length, 5 mm width, and 2 mm thickness. All tests were conducted the next day after printing at 22 °C. The samples were kept in a sealed bag in a dark place between printing and testing. The modulus values were obtained at 1 mm min^−1^. The maximal stress and strain values were measured at 5 mm min^−1^. The values for each material were determined by taking five parallel measurements.

##### DMA

The storage and loss modulus were determined as a function of temperature using a dynamic mechanical analyzer, a Mettler DMA/SDTA861e (Mettler Toledo, USA). The experiment used a dual cantilever deformation mode with a heating rate of 3 °C per minute in a temperature range of −70–100 °C, an applied force of 10 N, a displacement of 0.2 mm, and a frequency of 1 Hz. The sample size was 8.5 × 4 mm, with a thickness of 200 μm (four layers). The samples were kept in a sealed bag in a dark place between printing and testing.

The shape memory effect was analyzed using DMA Q800 (TA instruments, USA) in a typical shape memory testing cycle (controlled force mode). First, all samples were heated to *T*_g_ + 10 °C and kept at this temperature for 5 min, and then the force of 0.25 N was applied at an increasing rate of 0.05 N min^−1^. The sample was cooled down to *T*_g_‐60 °C and held for 5 min, after which the force was removed to a minimum force of 0.001 N by −0.05 N min^−1^ increments. For samples with *T*_g_‐60 values above 0 °C, the temperature was fixed at 0 °C due to machine limitations. For each sample 6 × 5.3 × 0.2 mm (*l* × *w* × *t*), the experiment was repeated for three cycles to achieve average shape recovery (*R*_r_) and shape fixity (*R*_f_) ratios, using Equation (2) and (3).

(2) $$R_{r}\left(N\right)=\frac{\varepsilon_{m}\left(N\right)-\varepsilon_{p}\left(N\right)}{\varepsilon_{m}\left(N\right)-\varepsilon_{p}\left(N-1\right)}\times 100$$

(3) $$R_{f}\left(N\right)=\frac{\varepsilon_{u}\left(N\right)}{\varepsilon_{m}\left(N\right)}\times 100$$

where *ε*_m_ is the strain in a temporary stretched state after cooling with force applied, *ε*_p_ is the strain after shape recovery, *ε*_u_ is the strain in the fixed temporary shape after cooling without force applied, and *N* is the cycle number. This approach closely simulates real‐life scenarios for SMP applications.

It should be noted that 0.25 N force was applied for the samples A_I1, A_I1M1, and A_I1M3, and 0.18 N for all remaining samples. The A_I1M7_0.1 and A_I1M7_0.2 were no*t* tested due to unoptimal mechanical performance.

##### Heat Conductivity

Thermal conductivity measurements were performed using the Netzsch laser flash method with the LFA 447 NanoFlash (Netzsch, Germany) according to EN ISO 22007‐4. Samples were printed in a square 12.7 × 12.7 × 0.9 mm shape. For measurements, samples were coated with graphite to ensure equal opacity and absorbance of samples. The heat conductivities of samples were obtained by subjecting all samples to five parallel thermal conductivity measurements at temperatures from 20 to 100 °C.

##### Electrical Resistance and Thermal Imaging of Joule Heating Effect

When a voltage is applied to a conductive polymer composite, the material generates heat through Joule heating (resistive heating). The overall conductivity of the sample depends not only on its dimensions and the applied voltage but also on its temperature, as resistance in most materials varies with temperature. This heating approach can induce the transition of the developed samples from a temporary shape to their permanent shape through a thermally driven transformation.^[^ ^16^ ^]^ An experimental setup was constructed to determine the voltage required to trigger this transition. The setup included a variable DC power supply operating in constant voltage mode, connected in series with an ammeter (Keithley 2001 Benchtop multimeter) to monitor changes in current over time and a FLIR E75 Thermal Imaging Camera—used to measure the temperature and monitor the temperature distribution on the sample's surface. A contrast between the samples and the background was achieved by using a cooler background than room temperature. The applied voltage was increased in 10 V increments every 3 min until the sample reached 100 °C. The obtained data was used to measure the electrical resistance of 3D printed 30 × 10 × 1 mm samples, indicating a negative temperature coefficient for all developed samples. Three parallel measurements for each sample were made. For the most optimal composition (A_I1M7_0.2_T), an additional test was carried out to determine the heating time. The sample was heated to a previously determined maximal temperature at a fixed voltage, and the thermal camera recorded the heating time. Then, the sample was let to cool down to room temperature, and a higher voltage was applied. Voltage was increased in 10 V increments.

##### Thermal Imaging of Light Heating Effect and 4D‐Printed SMP Structures Actuation with Light and Electricity

A PIR uc 605 camera with a wide angle lens (focal length of 10 mm and a FOV of 59° × 46°), 640 × 480 pixel picture resolution, and a frame rate ranging from 2.5 to 12.5 fps was used to capture infrared videos of the light heating effect and 4D‐printed structures. Due to variations in emissivity values, focusing was done by hand using a ruler with distinguishing markings. Contrasting the samples and the background by using a cooler background than room temperature was possible.

The light heating effect was studied using a 90 × 5 × 1 mm 4D‐printed bar irradiated sequentially at its center with an 808 nm laser (Cobalt IR 06 MLD) at powers ranging from 10 to 110 mW. After each irradiation, the sample was allowed to cool to room temperature before increasing the laser power. CNTs efficiently absorb near‐infrared light and convert it into thermal energy, causing the materials to heat up.^[^ ^65^ ^]^ This localized heating can induce a precisely controlled shape recovery through a thermally driven transformation similar to Joule heating.

The air actuation of 4D‐printed structures was achieved using a Ryobi hot air blower gun (EHG2020LCD). The heat gun was utilized in low‐intensity mode to prevent the sample from moving due to air movement, and a temperature threshold was established for each sample individually. The threshold was set to 60 °C for the A_I1M1 sample, 80 °C for the A_I1M7 sample, and 90 °C for all remaining samples.

To visualize the mechanical response of a double material spiral, the sample was heated to around 52 °C, and a 130 g hollow metal cube was placed on top for a short time to distort the spiral.

A Delta elektro (SM‐300‐5) power source was used at 60 V for the electrical actuation for all samples. For the actuation of the spiral structure, copper wires were attached to the top and bottom using silver paste for improved contact. For the actuation of conductive auxetic structure, copper wires were attached similarly at the desired positions, as shown in Figure 5a, dividing the structure into three segments. The voltage was sequentially applied to every segment until full shape recovery, changing the attached wires after every actuation. For the initial shape programming, the sample was secured in the experimental setup, and a 50 g weight was attached to its lower section. The sample was then heated above *T*_g_, causing elongation due to matrix softening, with the extension limited to 100 mm. Subsequently, the structure was cooled, allowing the new shape to be fixed.

For light actuation of the 4D printed 90 × 5 × 1 mm bar, first, it was heated above *T*_g_ and manually deformed to a random shape. Then, the laser was pointed at the desired position of the deformed sample until movement was observed on the recording. The laser power was set to 110 mW.

##### Measurement of Displacement Using Thermal Recordings

A custom Python script was used to process and analyze thermal video recordings. The script tracked user‐specified regions of interest over time, calculated distances between these regions of interest (ROIs), and generated both visual and quantitative outputs. Key parameters required by the script were stored in a configuration file. Within this file, tracking defines the ROIs in the format (*x*, *y*, width, and height); tmin_tmax describes the colormap temperature range used in the video analysis; search_val specifies the allowable search region to minimize abrupt positional shifts in ROI detection; crop removes superfluous background regions not relevant to the calculations; pos_temp: indicates which tracked points are used for median temperature calculations; and other parameters are no longer in use.

The script workflow consisted of the following major steps: 1) parameter loading; 2) image preprocessing (in a loop over each frame); 3) ROI tracking and update of coordinates; 4) distance calculation; 5) temperature calculation; and 6) visualization and output generation (end of loop).

Each incoming frame was normalized and denoised: Normalization was done by: cv2.normalize(frame, None, 0, 255, cv2.NORM_MINMAX, dtype = cv2.CV_8U). This step converted the original temperature values to a scaled 8‐bit range. Denoising was done by: cv2.fastNlMeansDenoising(frame_convert, None, *h* = 10, templateWindowSize = 5, searchWindowSize = 11). Denoising smoothed out background noise and enhanced the detection of sample edges.

For distance calculations, two points were tracked to measure the height of the target structure. When structures were tilted, the calculation considered the central positions, using only the *y*‐coordinates to compute distances. For each pair of ROIs:

Distances *D* were taken as differences in *y*‐coordinates (in pixels). The script saved the raw distance values and plotted the relative displacement, using the following.

(4) $$\frac{D-D_{0}}{D_{0}}\times \;100\text{ \%}$$

For temperature extraction, after cropping each frame around the tracked edges thresholding (cv2.threshold with Otsu's method) was applied to isolate the structure of interest from the background. A median temperature was calculated only from the thresholded (sample) pixels, excluding any surrounding background. This approach allowed reliable tracking of ROIs and the extraction of distance and temperature data throughout each thermal video recording, providing the basis for subsequent analyses and visualizations.

##### Statistical Analysis

Tensile tests were performed on five parallel samples. Resistivity and Joule heating were measured over three identical samples for each data point. All data are expressed as mean ± SD. All the data analyses were carried out using Excel. Representative shape memory performance tests with thermal imaging were performed with one sample.

## Conflict of Interest

The authors declare no conflict of interest.

## Author Contributions

**Maksims Jurinovs**: conceptualization (lead); data curation (lead); formal analysis (lead); funding acquisition (lead); investigation (equal); methodology (lead); validation (lead); visualization (lead); writing—original draft (lead); and writing—review and editing (lead). **Madara Veseta**: formal analysis (supporting); investigation (equal); and writing—original draft (supporting). **Alise Sabalina**: formal analysis (equal) and investigation (equal). **Pedro E. S. Silva**: data curation (supporting); investigation (equal); software (lead); validation (supporting); and writing—original draft (supporting). **Artis Linarts**: investigation (supporting); validation (supporting); and writing—original draft (supporting). **Hossein Baniasadi**: investigation (equal); validation (supporting); and writing—original draft (supporting). **Jaana Vapaavuori**: conceptualization (supporting); formal analysis (supporting); validation (supporting); and writing—review and editing (equal). **Sergejs Gaidukovs**: conceptualization (supporting); funding acquisition (supporting); project administration (supporting); supervision (supporting); and writing—review and editing (equal).

## Supporting information

Supplementary Material

## Data Availability

The data that support the findings of this study are available from the corresponding author upon reasonable request.
