# Supplementary material for: Sustainable 4D Printable Biobased Shape Memory Polymers with Linear Tunability and Multistimuli Actuation for Advanced Applications
Source: Small Sci. 2025 Apr 29;5(7):2500104. doi: 10.1002/smsc.202500104 (PMC12257905; doi:10.1002/smsc.202500104)
Supplement: Supplementary file 1 — Supplementary Material [file SMSC-5-2500104-s001.zip › smsc.202500104-sup-0001-suppdata-S1.pdf]

Sustainable 4D Printable Bio-Based Shape Memory Polymers with Linear Tunability and Multi-Stimuli Actuation for Advanced Applications

Maksims Jurinovs[1*], *Madara Veseta*[1], *Alise Sabalina*[1], *Pedro E. S. Silva*[2], *Artis Linarts*[3], *Hossein Baniasadi*[4], *Jaana Vapaavuori*[2], *Sergejs Gaidukovs*[1*]

[1] Institute of Chemistry and Chemical Technology, Faculty of Natural Sciences and Technology, Riga Technical University, P. Valdena Str. 3, LV-1048, Riga, Latvia
[2] Department of Chemistry and Materials Science, School of Chemical Engineering, Aalto University, Kemistintie 1, Espoo, 02150 Finland
[3] Institute of Physics and Materials Science, Faculty of Natural Sciences and Technology, Riga Technical University, Paula Valdena 3/7, Riga, LV-1048 Latvia
[4] Polymer Synthesis Technology, School of Chemical Engineering, Aalto University, Kemistintie 1, Espoo, 02150 Finland
Corresponding authors: Maksims.Jurinovs@rtu.lv; Sergejs.Gaidukovs@rtu.lv

Keywords: Actuators, Sustainability, Additive manufacturing, 3D printing, plant-based acrylates.

1. Results and Discussions

Table S1. Compositions of fully bio-derived 4D printable shape memory polymers and composites

Sample name	ARO, wt%	IBOA, wt%	IBOMA, wt%	AIBN, wt%	CNT, wt%
A_I1	50	50	0	-	-
A_I1M1	50	25	25	-	-
A_I1M3	50	12.5	37.5	-	-
A_I1M7	50	6.25	43.75	-	-
A_M1	50	0	50	-	-
A_I1M7_T	50	6.25	43.75	1	-
A_I1M7_0.1	50	6.25	43.75	-	0.1
A_I1M7_0.2	50	6.25	43.75	-	0.2
A_I1M7_0.2_T	50	6.25	43.75	1	0.2

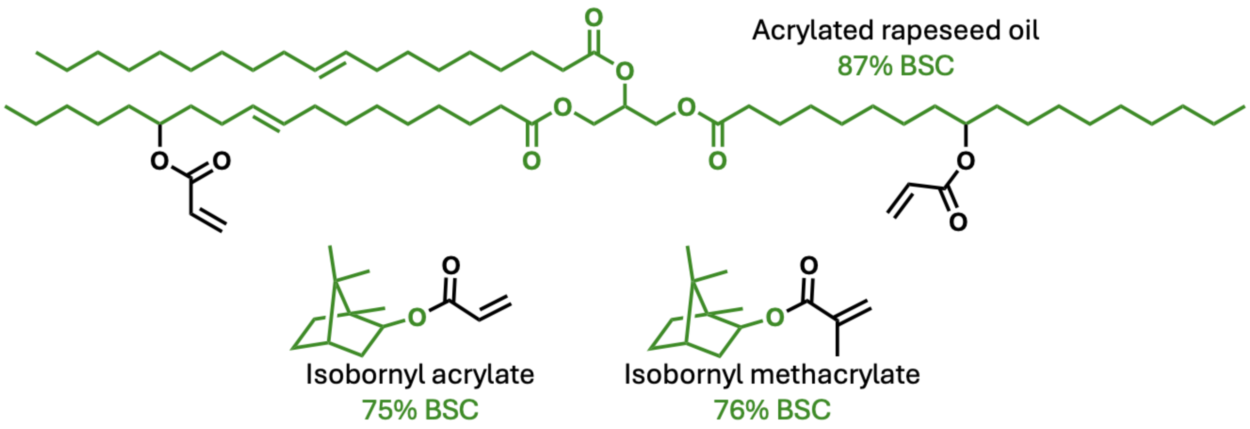

Figure S1. Structural formulas with bio sourced carbon (BSC) content of bio-derived acrylates used in the present study.

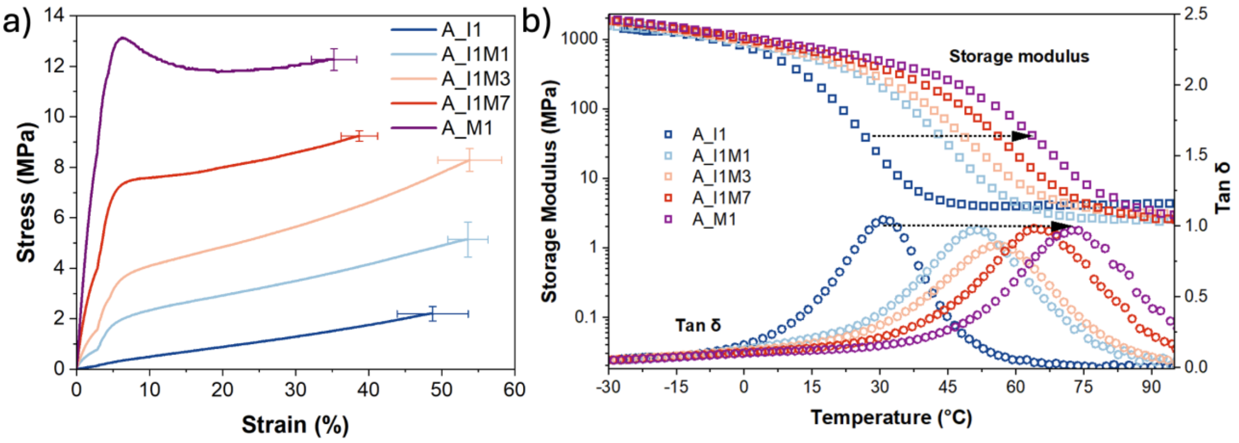

Figure S2. (a) Stress-strain curves, and (b) DMA storage modulus and Tanδ curves of 3D printed bio-derived polymers, with varying IBOA/IBOMA ratios.

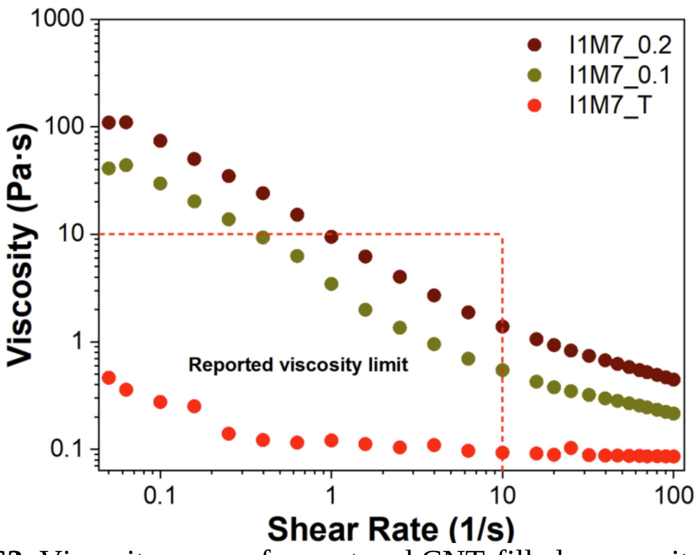

Figure S3. Viscosity curves for neat and CNT-filled composite resins.

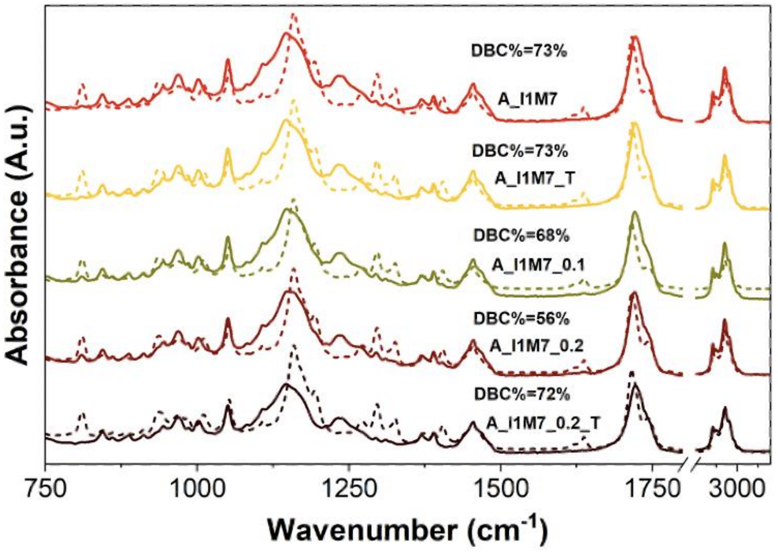

Figure S4. FTIR spectra before (dotted lines) and after (solid lines) UV-curing with calculated DBC% values of bio-derived CNT composites.

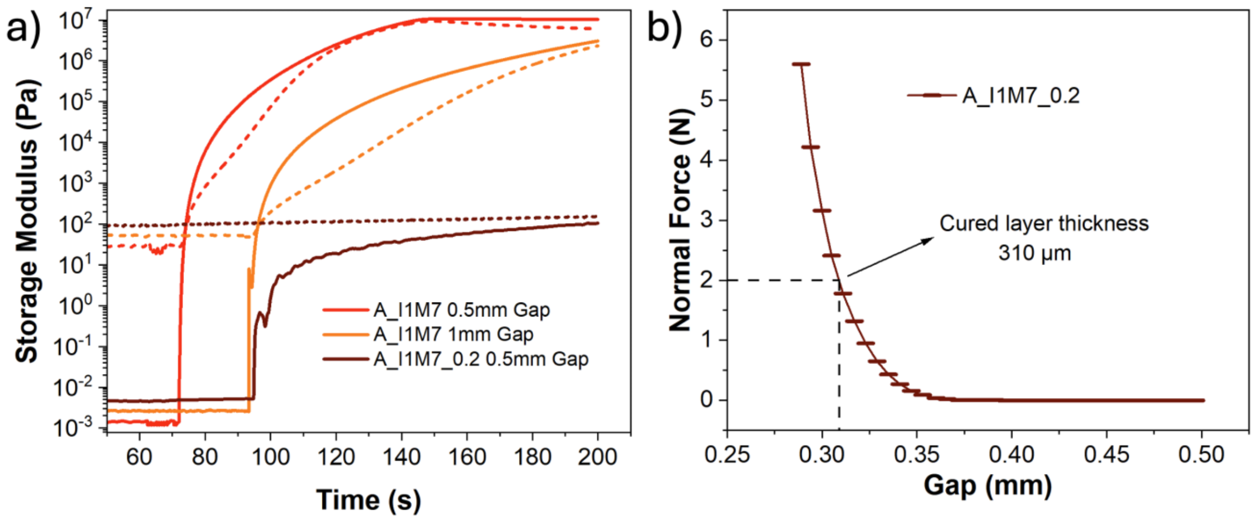

Figure S5. (a) Thick layer photo-rheology of A_I1M7 and A_I1M7_0.2, and (b) cured layer thickness of A_I1M7_0.2 over prolonged UV exposure time, showing reduced curing depth of CNT composite.

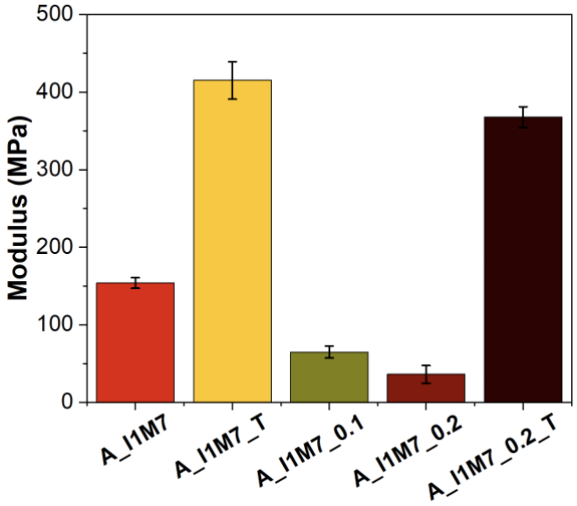

Figure S6. Tensile modulus of 4D printed bio-based SMP composite.

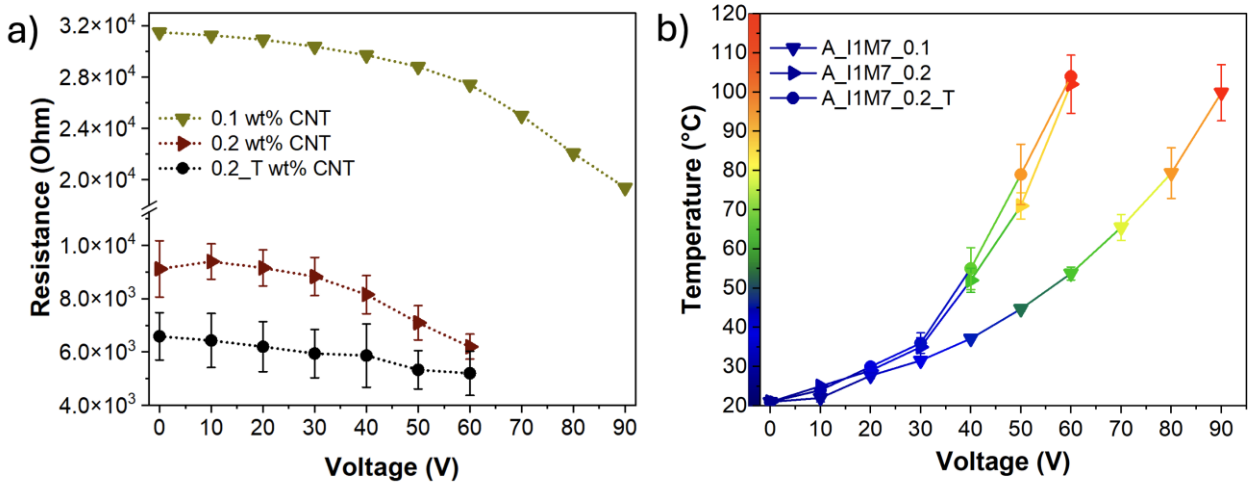

Figure S7. (a) Sample resistance as a function of applied voltage and (b) maximal achieved temperature of A_I1M7 composites with different CNT concentrations during Joule heating at different applied voltages.

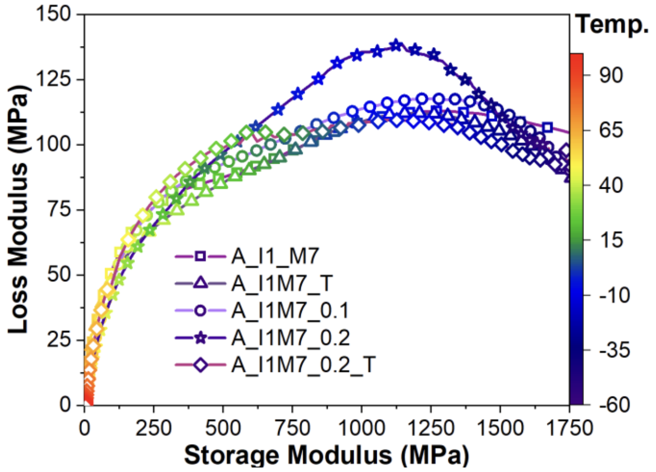

Figure S8. Cole-Cole plots of 4D printed chosen bio-derived polymer matrix and CNT composites with and without additional thermal post-curing.

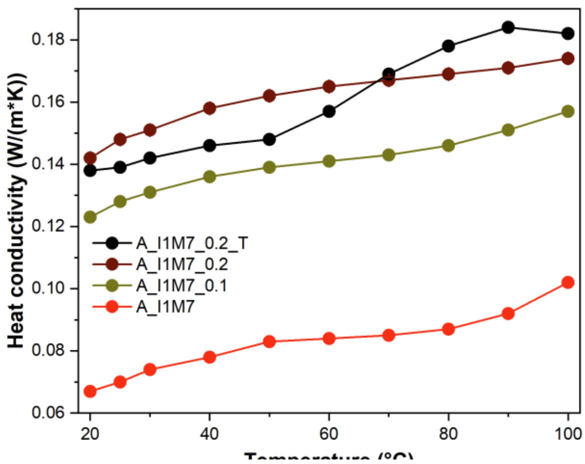

Figure S9. Sample heat conductivity as a function of temperature.

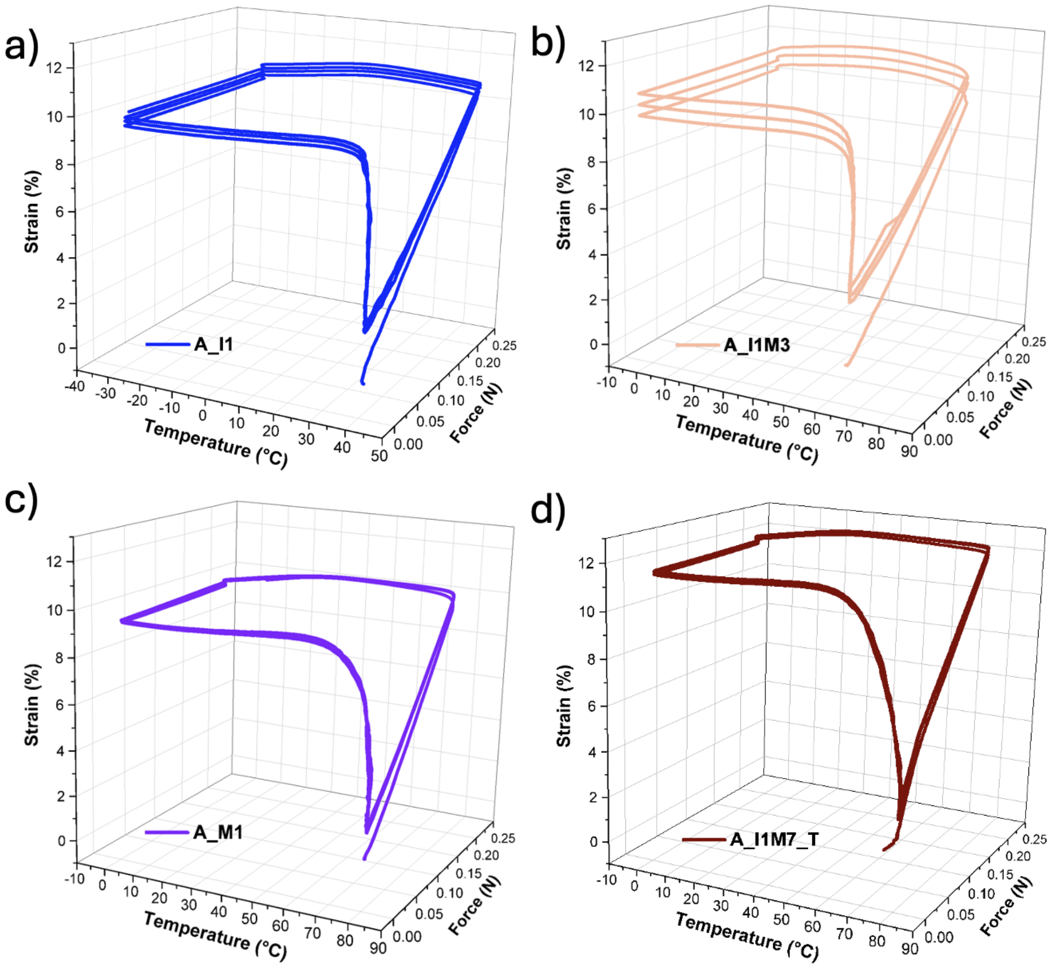

Figure S10. Shape memory effect DMA cycles for (a) A_I1, (b) A_I1M3, (c) A_M1 and (d) A_I1M7_T 4D printed films.

Table S2. Shape memory properties of fully bio-derived 4D printable shape memory polymer composites.

Sample name	Shape fixity	Shape Recovery
A_I1	98.81±0.09	97.38±0.55
A_I1M1	99.03±0.05	98.96±0.2
A_I1M3	98.73±0.02	96.6±0.35
A_I1M7	99.14±0.09	98.28±0.1
A_M1	98.31±0.55	98.56±1.16
A_I1M7_T	98.94±0.09	99.20±0.57
A_I1M7_0.1	-	-
A_I1M7_0.2	-	-
A_I1M7_0.2_T	98.21±0.01	98.87±0.62

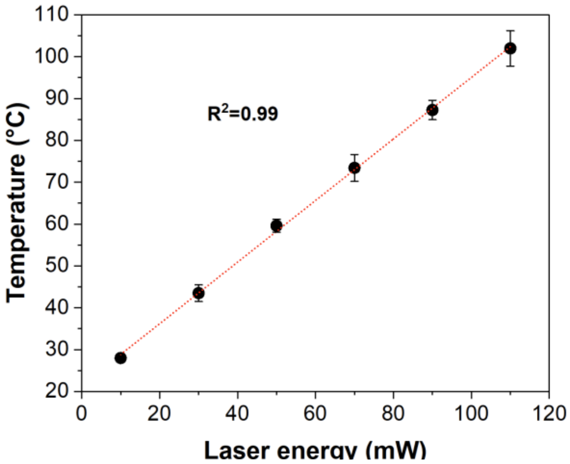

Figure S11. A_I1M7_0.2T composite temperature as a function of applied laser energy showing the linear relationship.

Video S1. Demonstration of the Joule heating effect showcasing the sample's temperature response to applied voltages ranging from 10 to 60 V.
Video S2. Demonstration of light-induced heating using an 808 nm IR laser, highlighting the sample's temperature response to applied energy levels ranging from 10 to 110 mW.
Video S3. Demonstration of the inability of the neat A_I1M7 4D-printed bio-derived matrix to be heated using an 808 nm IR laser at 110 mW.
Video S4. Comparison of the shape-memory effect in 4D-printed spirals fabricated with A_I1M1 and A_I1M7 bio-derived matrices. Differences in mechanical and thermomechanical properties influence activation temperatures and shape recovery speeds.
Video S5. Demonstration of distinct mechanical characteristics in a 4D-printed dual-material spiral. One half is printed with A_I1M1, and the other with A_I1M7, demonstrating differences in response to applied force.
Video S6. Sequential actuation of a 4D-printed dual-material (A_I1M1 and A_I1M7) spiral structure using hot air at different temperatures. The softer A_I1M1 section activates at ~50 °C, followed by the activation of the harder A_I1M7 section at higher temperatures.
Video S7. Sequential electrically initiated actuation of a 4D-printed A_I1M7_0.2_T composite auxetic structure at 60 V.
Video S8. One-step electrically initiated actuation of a 4D-printed A_I1M7_0.2_T composite spiral structure at 60 V.
Video S9. Comparison of one-step actuation between 4D-printed A_I1M7_0.2_T composite and neat A_I1M7 spiral structures using hot air.
Video S10. Sequential light-induced actuation of a 4D-printed A_I1M7_0.2_T bar at 110 mW 808 nm laser power.
